# Environmental disruption of host–microbe co-adaptation as a potential driving force in evolution

**DOI:** 10.3389/fgene.2014.00168

**Published:** 2014-06-20

**Authors:** Yoav Soen

**Affiliations:** Department of Biological Chemistry, Weizmann Institute of ScienceRehovot, Israel

**Keywords:** host–microbe symbiosis, genotype-to-phenotype transformations, canalization, developmental plasticity, epigenetics, dynamical systems, non-Mendelian inheritance, adaptation

## Abstract

The microbiome is known to have a profound effect on the development, physiology and health of its host. Whether and how it also contributes to evolutionary diversification of the host is, however, unclear. Here we hypothesize that disruption of the microbiome by new stressful environments interferes with host–microbe co-adaptation, contributes to host destabilization, and can drive irreversible changes in the host prior to its genetic adaptation. This hypothesis is based on three presumptions: (1) the microbiome consists of heritable partners which contribute to the stability (canalization) of host development and physiology in frequently encountered environments, (2) upon encountering a stressful new environment, the microbiome adapts much faster than the host, and (3) this differential response disrupts cooperation, contributes to host destabilization and promotes reciprocal changes in the host and its microbiome. This dynamic imbalance relaxes as the host and its microbiome establish a new equilibrium state in which they are adapted to one another and to the altered environment. Over long time in this new environment, the changes in the microbiome contribute to the canalization of the altered state. This scenario supports stability of the adapted patterns, while promoting variability which may be beneficial in new stressful conditions, thus allowing the organism to balance stability and flexibility based on contextual demand. Additionally, interaction between heritable microbial and epigenetic/physiological changes can promote new outcomes which persist over a wide range of timescales. A sufficiently persistent stress can further induce irreversible changes in the microbiome which may permanently alter the organism prior to genetic changes in the host. Epigenetic and microbial changes therefore provide a potential infrastructure for causal links between immediate responses to new environments and longer-term establishment of evolutionary adaptations.

## HYPOTHESIS

### THE MICROBIOME AS A ‘DOUBLE AGENT’ OF CANALIZATION AND DE-CANALIZATION

In his influential perspective on “Canalization and the inheritance of acquired characters” (Waddington, 1942), Conrad Waddington argued that evolution of developing organisms under natural selection tends toward canalization of the developmental process. That is to say, the sensitivity of developmental patterns to environmental and genetic perturbations is reduced over time in the environment to which the organism becomes adapted. A similar idea was developed independently by (Schmalhausen, 1949). Owing to this hypothesized tendency toward canalization, the patterns of development in the wild type organism are presumed to be more stable than the patterns in mutants (Waddington, 1942). This allows cryptic variation to be accumulated without phenotypic consequences. This variation can be unmasked by a sufficiently disruptive environmental or genetic perturbation (de-canalizing event) which breaks robustness and leads to phenotypic consequences reflecting the previously hidden variability (Flatt, 2005). Although canalization is most likely a systemic property, few specific molecular mechanisms have been proposed to support canalization [reviewed in (Salathia and Queitsch, 2007)]. These include buffering of genetic variability by the Hsp90 chaperone (Rutherford and Lindquist, 1998; Queitsch et al., 2002; Milton et al., 2006; Samakovli et al., 2007; Sgro et al., 2010), tissue-specific maintenance of active and repressed state of developmental genes by the Polycomb (and by analogy also the trithorax) system (Lee et al., 2005; Sawarkar and Paro, 2010; Stern et al., 2012), stabilizing negative feedback by microRNAs (Ronshaugen et al., 2005; Hornstein and Shomron, 2006; Li et al., 2009; Wu et al., 2009), and piwi-mediated silencing of transposon activity (Specchia et al., 2010; Gangaraju et al., 2011) or of existing variation (Gangaraju et al., 2011).

It is quite possible, though, that at least part of the noticeable contribution of these molecular pathways to canalization is due to their particularly wide range of interactions with other developmental genes and processes. In other words, these pathways are so deeply embedded into many regulatory feedbacks, that their disruption (by the environment or by a genetic change) can lead to significant impacts on developmental and physiological phenotypes. If correct, this rationale should hold more broadly for other intrinsic and extrinsic factors, which were co-opted to a particularly wide range of processes. One candidate factor is the microbiome which has co-evolved with its host for many generations. Similarly to host-intrinsic molecules and pathways that become involved in multiple functions, co-evolution of the microbiome with its host likely leads to integration of the microbiome into diverse host functions (Backhed et al., 2005; Rosenberg et al., 2009). This integration may be further widened and deepened by the ability of bacteria to produce many factors which could interact and cooperate with host processes. As part of this cooperation, the microbiome can provide the host with metabolites and signaling molecules (Backhed et al., 2004; Arora and Sharma, 2011; Shin et al., 2011; Flint et al., 2012; LeBlanc et al., 2013), assist in harvesting these molecules (Yamanaka et al., 1977; Brune and Friedrich, 2000; Backhed et al., 2005; Wang et al., 2011), synthesize organic compounds that are passed on to the host (Cavanaugh et al., 2006; Dubilier et al., 2008), regulate the immune system [reviewed in (Round and Mazmanian, 2009; Kranich et al., 2011)], reduce the propensity for disease (Markle et al., 2013), protect against invaders [Fukuda et al., 2011; Wang et al., 2013; which may be outcompeted and displaced by the microbiome (Kamada et al., 2012)], affect lifespan (Brummel et al., 2004; Behar et al., 2008) and fecundity (Ben-Yosef et al., 2010), influence mating preference (Sharon et al., 2010a), and likely perform many other developmental stage-dependent or independent functions yet to be discovered. Such evolved cooperation can result in wide, reciprocal inter-dependencies between the host and its microbiome. Owing to this broad dependency of the host on its microbial partners, disruption of the microbiome can compromise normal host development and homeostasis (Littman and Pamer, 2011; Martin et al., 2013). Conversely, the intact microbiome could contribute to the stability of host processes. This presumption is supported by recent work in a variety of organisms which have begun to uncover increasing numbers of host pathways and processes that could be affected by disruption of the microbiome (Brummel et al., 2004; Sharon et al., 2010b; Shin et al., 2011; Storelli et al., 2011), including modulation of epigenetic gene control [Takahashi, 2014; e.g., methylation of Toll-like receptors in intestine cells (Takahashi et al., 2011)]. As in the case of mutations (Waddington, 1942), a disrupted microbiome does not necessarily de-stabilizes every phenotype in every stressful scenario. However, it is expected to promote significantly more cases of compromised phenotypic robustness compared to cases of increased robustness.

When the host and its microbiome co-evolve in a stable, or a frequently occurring environment, they become adapted not only to this environment, but also to one another. We refer to this state as coordinated adaptation. Deviations from this co-adapted state (in response to environmental, genetic, or epigenetic modifications) could create stressful conditions promoting reciprocal changes in the host and its microbiome. Stated differently, modifications in the previously adapted microbiome can change the internal environment of the host. This change in the internal environment could promote response in the host which, in turn, may affect the conditions for survival and growth of the bacteria, and so on. Coordinated adaptation is re-established if and when the overall responses reduce the stress to the host and its microbiome to a level no longer sufficient for driving further changes. This is analogous to the Le Chatelier’s principle in chemistry (Atkins and de Paula, 2002), namely: if a system in chemical equilibrium is subjected to a disturbance, it tends to change in a way that opposes this disturbance. Thus, the establishment of a coordinated adaptation of the host and its microbiome can be viewed as an extension of the Le Chatelier’s principle, from chemical systems to self-organization of living cells and organisms.

**FIGURE 1:**
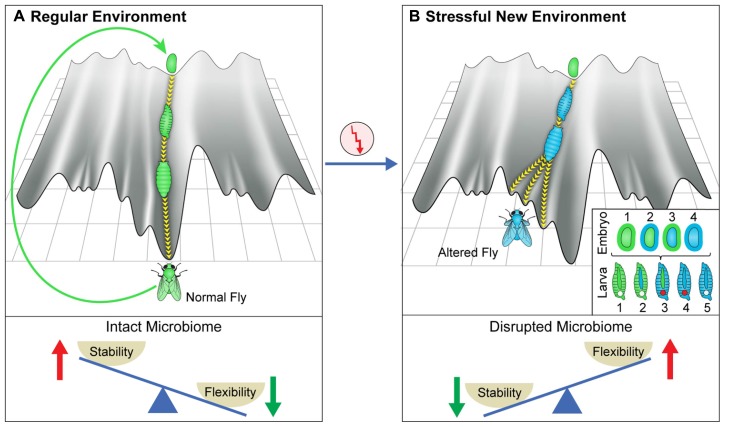
**Canalization and its disruption by stressful environments. (A)** Schematic depiction of the epigenetic landscape metaphor, viewing the process of development of an organism (e.g., fly) as a movement down a canal in epigenetic landscape. The landscape depends on the host genome and its environment, including its microbiome (which can be viewed as an internal environment). The canal represents the stage-dependent processes preventing deviations from the regular patterns of development in an environment to which the organism is adapted. The deeper the canal, the more robust are the patterns. The movement is perpetuated over generations by generating a fertilized egg positioned at the top of the same canal. **(B)** De-canalization by exposure to a stressful new environment. In this example, the change in the environment modifies the epigenetic landscape and increases the propensity of moving within a different canal. This can result in altered somatic tissues, altered germline and altered microbiome. Inset provides color-coded representation of various potential changes in the embryos and larval stages of a developing fly that has been exposed to a new stressful external environment. The gut microbiome is represented by the outer layer of the egg and the tube in the larva. The early embryo corresponds to the inner part of the egg and the germline is represented by the circle in larva. A change in color corresponds to alteration in the respective organ following the change in the external environment. **(Bottom)** Proposed, context-dependent influence of the microbiome on stability/flexibility. The microbiome contributes to stabilization in the regular environment to which the organism is well adapted (left). Disruption of the microbiome in a sufficiently stressful environment (right) destabilizes the host, thereby increasing phenotypic variability (increased flexibility).

Establishing a new co-adapted state involves interactions between the microbiome and the physiological and epigenetic state of the host. Important aspects of this interaction can be heuristically illustrated using the epigenetic landscape metaphor (Waddington, 1957). This metaphor views the development of an organism as movement down a canal (**Figure 1**, top left), starting with the fertilized egg and moving toward the adult form. Developed adults produce a fertilized egg positioned at the top of the same canal, thus starting the process over again. Here, the walls of the canal represent stabilizing (canalizing) processes, acting at every stage to prevent deviations from the normal patterns of development (termed by Waddington as homeorhesis to denote the constancy of flow along a determined trajectory). Still, the canal and the entire landscape depend on the host genome and its environment (Waddington, 1957), including the microbiome which can be viewed as an “internal” environmental factor. It is important to note, however, that the use of a 2-dimensional surface in this metaphor is an over simplification which may not properly represent attractors and flows in a high dimensional space corresponding to a developing organism. Moreover, because of potential influences of (past and present) environments and the dependence of these influences on the actual trajectory occupied by the organism, the details of the landscape are more dynamic and less pre-defined than often perceived.

The robust patterns of development in frequently encountered environments (to which the organism is adapted) correspond to movement in a particularly deep canal (although the depth of the canal is likely inversely correlated with the plasticity of the developmental process and may therefore be shallower during early specification of cells and body segments and possibly also during metamorphosis in which a given tissue type is transformed into another). At any given time in a frequently encountered environment, the microbiome “assists” the establishment of the adapted patterns and is therefore, an agent of canalization. However, since the epigenetic landscape can be influenced by the environment, a sufficiently strong environmental stress can modify the landscape to an extent sufficient to de-canalize the process of development OFF the “regular canal” and ON to a “side canal” (**Figure 1**, top right). The change in landscape can reflect a direct effect of the external environment on the host as well as an indirect effect through environmental disruption of the microbiome. Indeed, severe disruption of the microbiome could compromise the normal function of many host processes, including those which contribute to robustness. This would tend to destabilize (de-canalize) the adapted patterns, leading to dysbiosis and increased phenotypic variability (wider sampling of the available epigenetic landscape). While such microbial disruption is not expected in frequently occurring environments to which the organism is well adapted, it can be readily occur under severe stressful environments in which the organism is considerably maladapted. Thus, the microbiome can contribute to stability in frequently encountered environments, while promoting variability in sufficiently stressful new environments (**Figure 1**, bottom panels).

### SPECIES-SPECIFIC RATES OF ADAPTATION CREATE HOST–MICROBE INCOMPATIBILITIES WHICH DISRUPT CO-ADAPTATION AND PROMOTE RECIPROCAL CHANGES

Although the host and its microbiome form a partnership of organisms which propagate as a community, members of this partnership exhibit species-specific differences in the rate, type, and magnitude of responses to the environment (Zilber-Rosenberg and Rosenberg, 2008; Rosenberg and Zilber-Rosenberg, 2011). Owing to differences in generation time, population size, and developmental constraints, the microbiome can adapt to new environments on time scales that are much faster than those of genetic adaptation in the host (Zilber-Rosenberg and Rosenberg, 2008). For example, the doubling time of bacteria is on the order of 1 h whereas the generation time of a human host is about 20 years. Thus, within a single human generation, bacterial population can be propagated over 10^5^ generations. Combining this large number of generations with very large population sizes [>10^13^ bacterial cells in human (Backhed et al., 2005)] provides a very substantial potential for bacterial adaptation and diversification within a single generation of the host. Notably, modifications in the microbiome include changes in gene sequence (and/or function) within existing bacteria, and changes in species composition of the microbial community. This community often comprises many different species, whose relative abundance depends on the environment. As such, a stressful environment for the bacteria leads to species-specific selection which alters the composition and genetic structure of the community. This selection can be independent of whether or not the stress is also compromising the survival of the host.

**FIGURE 2:**
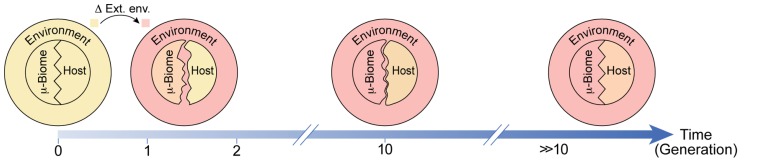
**Schematic of potential environmental influence on adaptation of the host and the microbiome.** Adaptations following a transition from the regular environment (light yellow) to a stressful novel environment (pink) are represented as matching of colors and shapes. Prior to the transition, the host and its microbiome are adapted to the environment (same color) and to one another (matched patterns). Shortly after the transition, the host is only mildly changed while the microbiome is considerably more adapted to the new environment (closer in color to the new environment). This difference in responsiveness disrupts the compatibility between the host and its microbiome (unmatched patterns). With time, the host and its microbiome become progressively more adapted to the environment and to one another.

**FIGURE 3:**
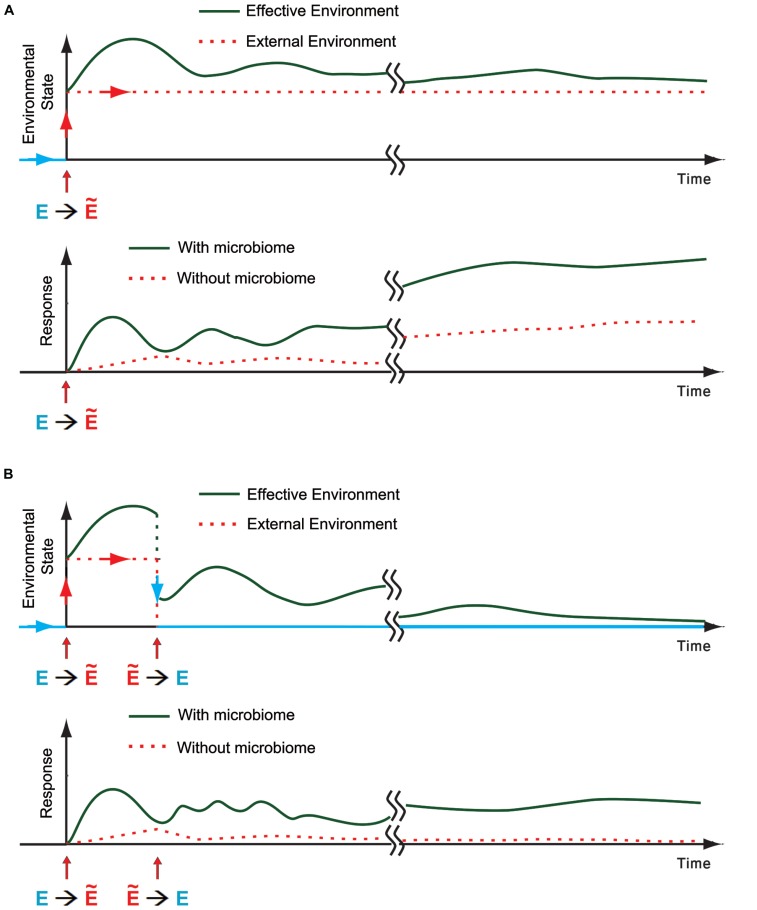
**The microbiome as an environmental component for the host. (A) Top:** Schematic representation of a transition from the regular external environment toward a new stressful environmental state (E→Ẽ). The effective environmental state (green) is the sum of all the environmental influences on the host, taking into accounts the external conditions and internal inputs from the microbiome. An example of one possible trace of effective environmental state is shown in green. **Bottom:** Schematic representation of a potential host response with and without contribution of microbial disruption by the external environment (green and dotted red, respectively). Disruption of the microbiome can increase the response of the host, leading to a potentially faster and more pronounced response which is also more diverse and affects more individuals. **(B)** Same as **(A)** when the environment reverts back (Ẽ→E) within generation.

While the bacteria can undergo substantial adaptation to new environments already within a single or few generations of the host, the latter remains largely genetically adapted to the previous environment (**Figure 2**). This can compromise the adaptation of the host to the new environment as well as its compatibility with the microbiome, leading to several distinct effects: first, disruption of the microbiome can destabilize the host, thereby enhancing its phenotypic responsiveness to the new environment (potentially resulting in increased occurrence, magnitude, and diversity of the phenotypic response). Additionally, the change in the microbiome is itself an “internal” environmental input which feeds back on the physiological and epigenetic response of the host (**Figure 3**). For example, the microbiome can respond to a new stress by producing (or elevating) a substance that is toxic to the host. This substance adds to the external stress and can modify the type and magnitude of the response in the host even if the external environment is back to normal (**Figure 3B**). This is because the microbiome may still remain altered and disruptive to the host. Thus, the response of the host to microbial-mediated inputs can persist as long as the host–microbe incompatibility is large enough to modify the host.

### TRANSGENERATIONAL IMPLICATIONS OF THE DISRUPTION OF COORDINATED ADAPTATION

The presumed contribution of the microbiome to the stabilization of host development and physiology is analogous to that of other factors (e.g., Hsp90) which can confer robustness to the organism in frequently occurring environments. However, unlike many other factors, the change in composition and/or genetic sequence of resident bacteria might itself be heritable, thereby providing potential infrastructure for multi-generational influences on the host (Zilber-Rosenberg and Rosenberg, 2008). Indeed, the microbiome in many organisms has been shown to be transmitted across generations either vertically or horizontally (Bright and Bulgheresi, 2010). Vertical transmission refers to transfer within the zygote, while horizontal transmission is based on various forms of pickup of micro-organisms from the surroundings, followed by their re-colonization within the host. These mechanisms of transmission normally facilitate inheritance of the intact (co-adapted) microbiome. However, the same mechanisms could support inheritance of environmentally modified microbiome. In line with this possibility, we have recently found that environmentally induced changes in the composition of gut bacteria in flies can be inherited (Fridmann-Sirkis et al., 2014). Similar findings are expected whenever the microbiome is altered to a degree which no longer supports full recovery within one generation. In this case, the offspring inherit altered microbiome which may or may not have been partially restored. The extent of inherited changes depends on the details of the external environment (type and strength of stress, its onset and duration, proximity to unaffected individuals, etc.), the internal conditions inside the host and the dynamics within the community of microbial species.

**FIGURE 4:**
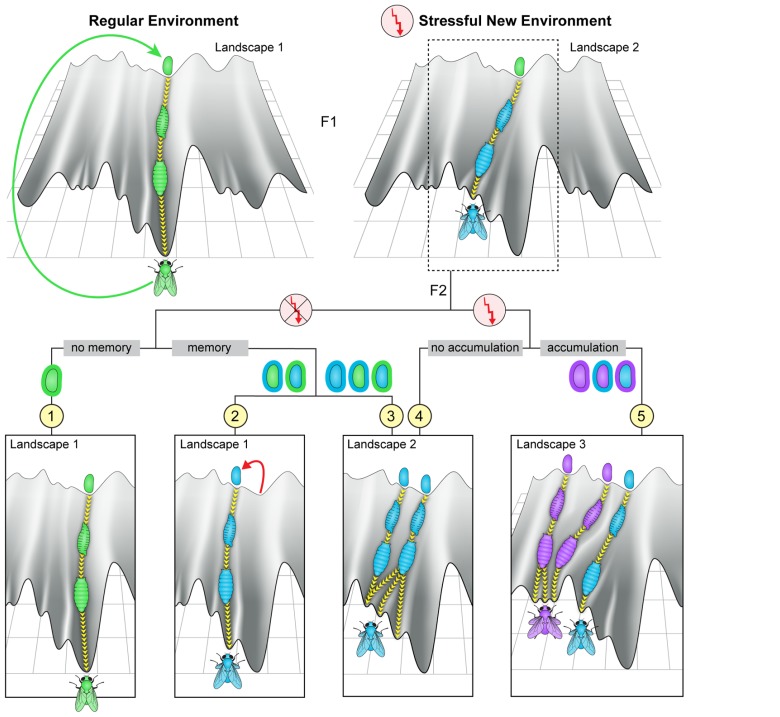
**Potential transgenerational impacts of environmental exposures. Top:** Heuristic depiction of fly development in an unperturbed landscape (left) and in a landscape modified by exposure to a strong environmental stress (right). The flow in a modified canal leads to characteristic modifications in somatic tissues of the fly and/or its germline and/or its microbiome. **Bottom:** Illustration of a few possible scenarios of development in subsequent offspring of exposed ancestors. Scenario (1): if the parental change in the environment does not modify the genome or the epigenetic state of the fertilized egg and, in addition, the microbiome is either unperturbed or fully restored (“no memory,” most left panel), the offspring may follow the regular trajectory in an unperturbed landscape. Scenarios (2 and 3): if the initial state of the fertilized egg and/or the microbiome associated with the egg are modified following the parental exposure (“memory”), the development of the offspring may differ from their parents even if the external environmental reverts to normal already during the parental generation. In these cases, alteration in offspring development might reflect localization to a different canal in the original or in a modified landscape (scenarios 2 and 3, left trajectory), or localization to a canal that has been modified by the impact of having modifications in the fertilized egg and/or the microbiome (scenario 3, right trajectory). Scenarios (4 and 5): if the external environmental stress persists beyond the parental generation it may again lead to changes in the entry point and shape of the canal (“no accumulation,” scenario 4) as well as further modify the entry point and shape (“accumulation,” scenario 5).

Modifications in the inherited microbiome, can change the initial conditions of offspring development. Depending on the structure and dynamics of the epigenetic landscape, this could correspond to a change in trajectory leading to a different outcome in the adult (**Figure 4**, top right). The altered adults could again produce modified embryos, thus enabling multi-generational changes in movements in epigenetic space. These multi-generational changes may involve different scenarios which could support non-Mendelian inheritance. Heuristic representation of some of these scenarios is depicted in the bottom panels of **Figure 4** (scenarios 2–5). For example, a change in the microbiome in one generation can potentially modify the host germline, leading to altered embryonic development of the offspring (viewed either as an altered initial point in the landscape or as movement in a modified landscape). Alternatively, persistent change in the microbiome can alter later stages of development, leading again to a modified offspring adult with a potentially modified microbiome. Evidence supporting potential influence of resident bacteria on the germline in insects has been reported for the endosymbiont *Wolbachia* (Werren, 1997; Stouthamer et al., 1999; Starr and Cline, 2002; Negri et al., 2006, 2009). More recent evidence in flies (*Drosophila melanogaster*) provided indirect evidence for influence of gut microbiome on the germ line (Fridmann-Sirkis et al., 2014). Specifically, removal of gut bacteria in one generation led to a substantial delay in larval development starting in the following generation. The phenotypic difference between parents and offspring following the depletion of gut bacteria (in parents) suggests an influence of these bacteria on the parental germline. Such a transgenerational effect of bacterial depletion was further shown to be responsible for the inheritance of a delay in larval development following parental exposure to G418 antibiotic (Fridmann-Sirkis et al., 2014). In this case, the delay in the first generation of larvae is caused by a direct toxicity of G418 in the host tissue, but is maintained in the non-exposed offspring by the transgenerational impact of depleted gut *Acetobacter* species in the G418-exposed parents. This unexpected situation is just one example of potentially diverse circumstances in which disruption of the microbiome could contribute to non-Mendelian transfer of influences. Thus, regardless of whether the effects of microbial changes are restricted to the soma or not, inheritance of a modified microbiome provides substantial infrastructure for multi-generational persistence of non-genetic changes in the host. This persistence depends on the strength and duration of the environmental stress, the degree of incompatibility between the host and the microbiome, and their inherent plasticity, or adaptability. The manner in which these factors are combined has a significant effect on the timescale of establishing a new equilibrium state.

### TIMESCALES OF INTERACTIONS BETWEEN STRESS-INDUCED EPIGENETIC AND MICROBIAL CHANGES

Current understanding of dynamical systems suggests that interaction between stress-induced disruption of the microbiome and modifications in the epigenetic state provide potential for a rich variety of dynamical outcomes. Indeed, coupling between relatively simple non-linear systems can generate diverse modes of collective or non-collective dynamics, depending on the intrinsic parameters of the uncoupled systems, the type and strength of the coupling, the type and magnitude of the non-linearity, and the initial conditions (Kuramoto, 1984; Glass and MacKey, 1988; Badola et al., 1991; Otsuka, 1991; González-Miranda, 2004). For example, specific choices of couplings between two non-linear oscillators with sufficiently similar intrinsic frequencies can lead to synchronization of these oscillators, doublings of their intrinsic or collective periods, chaotic dynamics (of either one or both) and destruction of the oscillatory behavior (Sarkar et al., 2013). It is plausible that analogous wealth of dynamics could be generated by interaction between the microbiome and the epigenetic state of the host (regarded here in an inclusive sense which covers various types of non-genetic host-intrinsic changes). This interaction can be viewed as coupling between two non-linear systems, each modulating the activity of the other. Unlike the slowly varying genome sequence of the host, epigenetic changes in the host can occur on a range of timescales which overlaps with timescales associated with microbial changes. This provides potential for inter-modulations which prolong the duration of changes expected in a microbiome-free host. Encountering such scenarios under extreme forms of stress is quite plausible and may have occurred in a recent example of transgenerational inheritance following parental exposure to toxic stress (Stern et al., 2012). This inheritance was shown to involve host-intrinsic (Stern et al., 2014) and microbial-mediated mechanisms (Fridmann-Sirkis et al., 2014), and typically persisted for 3–10 generations. It is possible that interactions between the host-intrinsic and microbial-mediated modifications extended the duration of inheritance beyond the duration that would have been observed without an interaction.

Depending on the duration of change in the external environment, one can further distinguish three different regimes:

#### Stressful external conditions which persist for only one, or a small number of generations

This regime of stress may initiate reciprocal modifications in the microbiome and the host. For most stressful environments, the modifications in the host would be primarily non-genetic (e.g., physiologic, metabolic, epigenetic etc.’). The microbiome, on the other hand, may change in gene sequence and species composition (in addition to epigenetic changes). Owing to the heritability of some of these changes [and potentially also heritability of epigenetic changes in the host (Jablonka and Raz, 2009; Jablonka, 2013; Lim and Brunet, 2013)], return to the normal environment will not necessarily lead to immediate reversion of the host back to its previous state (**Figure 4**, panels 2 and 3). This is because reversal of the environment does not necessarily eliminate host–microbe incompatibilities (and/or persistent host-intrinsic epigenetic changes) that have been induced during the external stress period. The induced host–microbe incompatibility promotes cross talk between the microbiome and the physiologic/epigenetic state of the host, eventually leading to establishment of a new co-adapted state in which the host and its microbiome become re-adapted to one another. The process of relaxation toward an altered state could span multiple generations of the host, but might be counteracted by re-exposure to non-modified microbiome (e.g., by infection-mediated transfer from individuals which were not exposed to the new environmental conditions).

#### Persistence of the stressful new environment over timescales much longer than required to reach a new host–microbe equilibrium state

This scenario likely promotes more extensive and longer lasting changes in the host and the microbiome. After a few generations in this stable environment, the microbiome will likely become irreversibly modified. The modified microbiome will continue to change with its host until they become sufficiently adapted to the altered environment and to one another. If, in addition, the stressful environment influences a large geographic region, it will substantially reduce the chances of encountering non-exposed individuals, thus reducing the chances of infection-mediated reversion to the original microbiome. It is also possible that the microbiome niche will be taken over by modified bacteria that are abundant enough to prevent re-colonization of the original bacteria. Additionally, the host may become sufficiently modified so as to favor the altered microbiome over the original microbiome.

#### Persistence of external stress over a time period comparable to that required for co-adaptation

In this intermediate case, equilibrium of the host and the microbiome can be re-challenged by the reversion of the environment, thus prolonging the establishment of a new stable state. Since the timescales of environmental change might indeed be comparable to those of epigenetic and microbial changes, this scenario may not be extremely rare. Additionally, the likelihood of encountering this scenario may increase when the organism is capable of modifying its external environment (niche construction).

Taken together, the above regimes support phenotypic diversification and/or adaptation over timescales ranging from few to many generations. This diversification may become irreversible prior to genetic changes and adaptation in the host. As such, it may provide important bridge between the physiological timescales of one generation and the much longer timescales of genetic adaptation and phylogenetic diversification.

**FIGURE 5:**
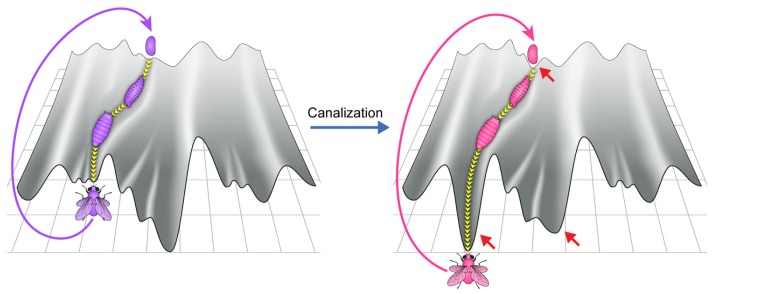
**Heuristic depiction of increased canalization over time in a new environment (right vs. left panel).** The process of development becomes confined to a deeper and less rugged canal, reflecting, respectively, increased developmental robustness and reduction in the number of developmental outcomes. Red arrows indicate expected changes in the landscape.

According to the original canalization hypothesis (Waddington, 1942; Schmalhausen, 1949), a new co-adapted state of the host and its microbiome is expected to become progressively more canalized (stabilized) over time (**Figure 5**). The principles and mechanisms leading to progressive stabilization are not entirely clear. It was suggested that the trend toward stabilization is implemented by increasing the connectivity of the underlying regulatory network, which is in turn supported by a tendency of natural selection to favor stable development (Siegal and Bergman, 2002). An increase in regulatory complexity can be achieved by different means, including by wider integration (co-option) of the microbiome into host processes. Increasing integration may reflect an entropic effect which is expected from having many more potential scenarios of integration compared with no integration. Among these scenarios would be host–microbiome integrations which either increase or reduce the stability of development. However, if changes which increase the stability tend to be more persistent, the entropic force of increasing co-option is expected to promote progressively more extensive host–microbiome interactions that are biased toward increased stability.

### DIFFERENT UNITS OF SELECTION AND THEIR IMPLICATIONS FOR ADAPTATION AND EVOLUTION

Much of the above considerations stems from having more than one unit of selection. First, the host and its microbes are subjected to selection as a cooperating community. This is the most relevant unit of selection for the host. On the other hand, each of the resident bacterial species is subjected to its own selection conditions within the host. The outcomes of these individual selections feed into the higher level of host (or community) selection.

This interaction between different layers of selection has far reaching implications which are not accounted for in current models of population genetics (Jaenike, 2012). The latter often consider changes in allele frequencies in the host, ignoring the potential contribution of changes in bacterial allele frequencies to the selection of the host. These changes in the microbiome could affect the propagation of the host, either by modifying it or by changing its internal environment. Owing to these changes, the host phenotypes and epigenetic state may be stably altered prior to the emergence or fixation of adaptive genetic changes in its own genome.

Selection of bacterial species within the host may also promote maladaptive changes in the host, thus providing potential for conflicts between the microbiome and the host. On longer timescales, however, these conflicts are subjected to selection at the higher, community level, which could then promote successful communities, and hence, successful hosts (Zilber-Rosenberg and Rosenberg, 2008). A somewhat similar, host-intrinsic scenario involving multiple layers of selection is provided by somatic mutations which lead to generation of tumor cells. These cells undergo changes promoting their own propagation on the expense of the host. However, unlike the microbiome, these tumors are not inherited, and do not evolve to cooperate with their host.

What is then the added evolutionary value of having these two layers of selection? Here, one can immediately recognize a few important considerations: first, the microbiome offers a substantial gene and cellular pool which can evolve to support reproduction of the community under new environmental conditions (Zilber-Rosenberg and Rosenberg, 2008). For example, functions and products of host cells may be provided as a service by resident bacteria (Backhed et al., 2005). This includes extreme cases in which endosymbiotic bacteria synthesize organic compounds (e.g., by chemosynthesis) and provide nutrition to certain gutless flatworms (Ott, 1982). The feasibility of such remarkable collaborations is indicative of the vast potential of the microbiome to generate “creative” solutions. Important components of this potential are the relative speed in which these solutions can emerge and the separation from the host. Having these rapid changes in entities distinct from the host allows the community to dramatically expand the range of genetic and cellular variation while paying significantly reduced costs of maladaptive changes. Indeed, a comparable extent of genetic and cellular changes in the host itself, will likely lead to failed development, or alternatively, tumorigenesis and death. In other words, variation of the microbiome is likely a safer option in much the same way as would therapy with bacteria as opposed to host-intrinsic gene therapy. Thus, the microbiome offers extended potential for rapid, flexible, and safer exploration of new solutions and functions which benefit the host. This potential is particularly important when the host encounters new problems which can be not eliminated fast enough by a change in the host, but may be alleviated by rapid adaptation of the microbiome. Thus, the microbiome may provide a developing organism with a substantial capability to adapt to novel stressful conditions. It is often assumed that the demand for such capability is not very strong because of the extremely low likelihood of encountering a truly novel environment. However, this rationale may not necessarily invalidate the demand for adaptive capacity because the organism may also face new stressful conditions in regular environments. Indeed, many changes in the genome, epigenome, or the microbiome can create stressful conditions. Owing to the immensely large space of possible changes at these levels, it is not be realistic to assume that a large fraction of all possible scenarios have been frequently encountered during the evolutionary history of the organism. In this case, the organism is not expected to be equipped with an efficient genetic program for coping with all these stressful scenarios. Thus, the demand for at least some ability to adapt to new conditions within one or few generations can be quite high. Future work will determine whether and how epigenetics and symbiosis provide the organism with such adaptive plasticity.

### DIRECTIONS FOR FUTURE STUDIES

The hypothesized involvement of the microbiome in regulating stability and flexibility based on contextual demand generates clear predictions which could be used to assess the validity of this hypothesis. In particular, the hypothesis asserts that: (1) intact microbiome tends to suppress environmentally- and genetically-induced phenotypic variation (compared to modified microbiome), and (2) severe stressful conditions tend to compromise this buffering by disrupting the composition and/or genetic and epigenetic makeup of the intact microbiome. These predictions can be tested by combining experimental manipulation of the microbiome with environmental and genetic perturbations. For example, the buffering capacity of the intact bicrobiome can be tested by comparing phenotypic variation in germ-free animals with variation in animals containing intact bacteria. Since the hypothesized buffering refers only to a statistical tendency to stabilize the phenotypes, the evaluation must be based on a sufficiently large set of experimental setups, involving different phenotypes in a variety of organisms subjected to different conditions (e.g., malnutrition, exposure to environmental stressors, use of genetically modified animals etc.). If the hypothesis is correct, we expect a positive correlation between lack of bacteria and the extent of deviation from regular developmental phenotypes. Positive correlation is also expected for milder manipulations of the microbiome, but the statistical validation in this case likely requires averaging across larger sets of conditions and phenotypes. It is also important to note that compromised (or complete lack of) microbiome in otherwise good conditions might not be disruptive enough to modify particular phenotypes in the host. Still, if a compromised microbiome reduces buffering capacity in the host, it should increase the likelihood that additional environmental or genetic insults will induce phenotypic variation. We therefore expect that the positive effect of bacterial disruption on the induction of phenotypic variability would tend to increase as a function of the overall amount of stress. The second part of the hypothesis (environmental suppression of phenotypic buffering by bacteria), can be tested by exposing developing organisms to different types of severe environmental stress and analyzing the ability of the stress to modify the overall composition of the microbiome as well as the properties of its individual bacterial species. Here again, the evaluation should include a variety of stress paradigms which do not involve stressors that have been specifically selected for targeting bacteria.

To further evaluate the hypothesized contribution of the microbiome to stable diversification and adaptation prior to genetic changes in their host, it would be instrumental to establish experimental setups in which the host is lacking defined functionalities. Combining these with multi-generational analysis of the type and stability of changes in the host and its microbiome should provide direct assessment of the validity of this hypothesis.

While the above evaluations should ultimately include a variety of different organisms, it may be useful to focus on organisms enabling rapid and rigorous assessment. One example is the fruit fly, *D. melanogaster*, which contains a relatively small number of (culturable) bacterial species (Wong et al., 2011). Additionally, the gut microbiome of the fly can be easily manipulated without relying on anti-microbial agents that could also have a direct impact on host tissues (Ridley et al., 2013). Avoiding these direct impacts is crucial for creating defined setups enabling rigorous evaluation of the influence of microbial changes on the host (Ridley et al., 2013; Fridmann-Sirkis et al., 2014). It is also important to devote attention to the potential impact of bacterial manipulations on the germline. Such impact can generate phenotypic differences between parents and offspring (Fridmann-Sirkis et al., 2014) thereby invalidating certain comparisons between non-matched generations. Avoiding these potentially confounding effects is achieved in flies by dissolving and sterilizing the chorionic shell of fertilized eggs (without antibiotics or propagation under germ-free conditions for an uncharacterized number of generations). Larvae that hatch from these dechorionated eggs are devoid of gut bacteria and can be re-exposed to defined compositions of bacterial species (e.g., bacteria that were isolated from intact flies with or without additional manipulations). These useful features synergize with the obvious benefits of having a large knowledgebase, powerful genetic tools and generation time which is compatible with practical analysis over about 10 generations.

The outcome of these (and similar) studies may shed important light on two critical questions in gene regulation and evolution, namely: (1) How the balance between stability and flexibility of developing organisms can be regulated by the environment based on contextual demand, and (2) Whether and how epigenetic- and symbiotic-based responses to new stressful conditions are causally connected to longer-term establishment of genetic adaptations in the host. This includes evaluation of the scope and mechanisms by which the adaptive potential of the microbiome might contribute to rapid buildup of stable adaptive modifications in the host. Following these lines of investigation may uncover hitherto unrecognized mechanisms bridging ecological and evolutionary processes.

## Conflict of Interest Statement

The author declares that the research was conducted in the absence of any commercial or financial relationships that could be construed as a potential conflict of interest.

## References

[B1] Arora T. Sharma R. (2011). Fermentation potential of the gut microbiome: implications for energy homeostasis and weight management. *Nutr. Rev.* 69 99–106 10.1111/j.1753-4887.2010.00365.x 21294743

[B2] Atkins P. W de Paula J. (2002). *Physical Chemistry* 7th Edn. Oxford: Oxford University Press

[B3] Backhed F. Ding H. Wang T. Hooper L. V. Koh G. Y. Nagy A. (2004). The gut microbiota as an environmental factor that regulates fat storage. *Proc. Natl. Acad. Sci. U.S.A.* 101 15718–15723 10.1073/pnas.0407076101 15505215 PMC524219

[B4] Backhed F. Ley R. E. Sonnenburg J. L. Peterson D. A. Gordon J. I. (2005). Host-bacterial mutualism in the human intestine. *Science* 307 1915–1920 10.1126/science.1104816 15790844

[B5] Badola P. Kumar V. R. Kulkarni B. D. (1991). Effects of coupling nonlinear-systems with complex dynamics. *Phys. Lett. A* 155 365–372 10.1016/0375-9601(91)91041-B

[B6] Behar A. Yuval B. Jurkevitch E. (2008). Gut bacterial communities in the Mediterranean fruit fly (*Ceratitis capitata*) and their impact on host longevity. *J. Insect Physiol.* 54 1377–1383 10.1016/j.jinsphys.2008.07.011 18706909

[B7] Ben-Yosef M. Aharon Y. Jurkevitch E. Yuval B. (2010). Give us the tools and we will do the job: symbiotic bacteria affect olive fly fitness in a diet-dependent fashion. *Proc. Biol. Sci.* 277 1545–1552 10.1098/rspb.2009.2102 20071385 PMC2871834

[B8] Bright M. Bulgheresi S. (2010). A complex journey: transmission of microbial symbionts. *Nat. Rev. Microbiol.* 8 218–230 10.1038/nrmicro2262 20157340 PMC2967712

[B9] Brummel T. Ching A. Seroude L. Simon A. F. Benzer S. (2004). *Drosophila* lifespan enhancement by exogenous bacteria. *Proc. Natl. Acad. Sci. U.S.A.* 101 12974–12979 10.1073/pnas.0405207101 15322271 PMC516503

[B10] Brune A. Friedrich M. (2000). Microecology of the termite gut: structure and function on a microscale. *Curr. Opin. Microbiol.* 3 263–269 10.1016/S1369-5274(00)00087-4 10851155

[B11] Cavanaugh C. M. McKiness Z. P. Newton L. G. Stewart F. J. (2006). “Marine chemosynthetic symbioses,” in *The Prokaryotes* Vol.1 *Symbiotic Associations, Biotechnology, Applied Microbiology* edsDworkin M. Reosenberg E. Schleifer K.-H. Stackebrandt E. (New york: Springer-Verlag)

[B12] Dubilier N. Bergin C. Lott C. (2008). Symbiotic diversity in marine animals: the art of harnessing chemosynthesis. *Nat. Rev. Microbiol.* 6 725–740 10.1038/nrmicro1992 18794911

[B13] Flatt T. (2005). The evolutionary genetics of canalization. *Q. Rev. Biol.* 80 287–316 10.1086/432265 16250465

[B14] Flint H. J. Scott K. P. Louis P. Duncan S. H. (2012). The role of the gut microbiota in nutrition and health. *Nat. Rev. Gastroenterol. Hepatol.* 9 577–589 10.1038/nrgastro.2012.156 22945443

[B15] Fridmann-Sirkis Y. Stern S. Elgart M. Galili M. Zeisel A. Shental A. (2014). Delayed development induced by toxicity to the host can be inherited by a bacterial-dependent, transgenerational effect. *Front. Genet.* 5:27 10.3389/fgene.2014.00027 PMC3933808 24611070

[B16] Fukuda S. Toh H. Hase K. Oshima K. Nakanishi Y. Yoshimura K. (2011). Bifidobacteria can protect from enteropathogenic infection through production of acetate. *Nature* 469 543–547 10.1038/nature09646 21270894

[B17] Gangaraju V. K. Yin H. Weiner M. M. Wang J. Huang X. A. Lin H. (2011). *Drosophila* Piwi functions in Hsp90-mediated suppression of phenotypic variation. *Nat. Genet.* 43 153–158 10.1038/ng.743 21186352 PMC3443399

[B18] Glass L. MacKey M. C. (1988). *From Clocks to Chaos: The Rhythms of Life*. Princeton: Princeton University Press

[B19] González-Miranda J. M. (2004). *Synchronization and Control of Chaos – An Introduction for Scientists and Engineers.* London: Imperial College Press

[B20] Hornstein E. Shomron N. (2006). Canalization of development by microRNAs. *Nat. Genet.* 38(Suppl.) S20–S24 10.1038/ng1803 16736020

[B21] Jablonka E. (2013). Epigenetic inheritance and plasticity: the responsive germline. *Prog. Biophys. Mol. Biol.* 111 99–107 10.1016/j.pbiomolbio.2012.08.014 22975443

[B22] Jablonka E. Raz G. (2009). Transgenerational epigenetic inheritance: prevalence, mechanisms, and implications for the study of heredity and evolution. *Q. Rev. Biol.* 84 131–176 10.1086/598822 19606595

[B23] Jaenike J. (2012). Population genetics of beneficial heritable symbionts. *Trends Ecol. Evol.* 27 226–232 10.1016/j.tree.2011.10.005 22104387

[B24] Kamada N. Kim Y. G. Sham H. P. Vallance B. A. Puente J. L. Martens E. C. (2012). Regulated virulence controls the ability of a pathogen to compete with the gut microbiota. *Science* 336 1325–1329 10.1126/science.1222195 22582016 PMC3439148

[B25] Kranich J. Maslowski K. M. Mackay C. R. (2011). Commensal flora and the regulation of inflammatory and autoimmune responses. *Semin. Immunol.* 23 139–145 10.1016/j.smim.2011.01.011 21292499

[B26] Kuramoto Y. (1984). *Chemical Oscillations, Waves, and Turbulence*. Berlin: NY: Springer-Verlag 10.1007/978-3-642-69689-3

[B27] LeBlanc J. G. Milani C. de Giori G. S. Sesma F. van Sinderen D Ventura M. (2013). Bacteria as vitamin suppliers to their host: a gut microbiota perspective. *Curr. Opin. Biotechnol.* 24 160–168 10.1016/j.copbio.2012.08.005 22940212

[B28] Lee N. Maurange C. Ringrose L. Paro R. (2005). Suppression of Polycomb group proteins by JNK signalling induces transdetermination in *Drosophila* imaginal discs. *Nature* 438 234–237 10.1038/nature04120 16281037

[B29] Li X. Cassidy J. J. Reinke C. A. Fischboeck S. Carthew R. W. (2009). A microRNA imparts robustness against environmental fluctuation during development. *Cell* 137 273–282 10.1016/j.cell.2009.01.058 19379693 PMC2674871

[B30] Lim J. P. Brunet A. (2013). Bridging the transgenerational gap with epigenetic memory. *Trends Genet.* 29 176–186 10.1016/j.tig.2012.12.008 23410786 PMC3595609

[B31] Littman D. R. Pamer E. G. (2011). Role of the commensal microbiota in normal and pathogenic host immune responses. *Cell Host Microbe* 10 311–323 10.1016/j.chom.2011.10.004 22018232 PMC3202012

[B32] Markle J. G. Frank D. N. Mortin-Toth S. Robertson C. E. Feazel L. M. Rolle-Kampczyk U. (2013). Sex differences in the gut microbiome drive hormone-dependent regulation of autoimmunity. *Science* 339 1084–1088 10.1126/science.1233521 23328391

[B33] Martin R. Miquel S. Ulmer J. Kechaou N. Langella P. Bermudez-Humaran L. G. (2013). Role of commensal and probiotic bacteria in human health: a focus on inflammatory bowel disease. *Microb. Cell Fact. * 12:71 10.1186/1475-2859-12-71 PMC3726476 23876056

[B34] Milton C. C. Ulane C. M. Rutherford S. (2006). Control of canalization and evolvability by Hsp90. *PLoS ONE* 1:e75 10.1371/journal.pone.0000075 PMC1762401 17183707

[B35] Negri I. Franchini A. Gonella E. Daffonchio D. Mazzoglio P. J. Mandrioli M. (2009). Unravelling the *Wolbachia* evolutionary role: the reprogramming of the host genomic imprinting. *Proc. Biol. Sci.* 276 2485–2491 10.1098/rspb.2009.0324 19364731 PMC2690474

[B36] Negri I. Pellecchia M. Mazzoglio P. J. Patetta A. Alma A. (2006). Feminizing *Wolbachia* in *Zyginidia pullula* (Insecta, Hemiptera), a leafhopper with an XX/X0 sex-determination system. *Proc. Biol. Sci.* 273 2409–2416 10.1098/rspb.2006.3592 16928646 PMC1636090

[B37] Otsuka K. (1991). Complex dynamics in coupled nonlinear element systems. *Int. J. Mod. Phys. B* 5 1179–1214 10.1142/S0217979291000572

[B38] Ott J. A. (1982). New mouthless interstitial worms from the sulfide system: symbiosis with Prokaryotes. *Mar. Ecol.* 3 313–333 10.1111/j.1439-0485.1982.tb00282.x

[B39] Queitsch C. Sangster T. A. Lindquist S. (2002). Hsp90 as a capacitor of phenotypic variation. *Nature* 417 618–624 10.1038/nature749 12050657

[B40] Ridley E. V. Wong A. C. Douglas A. E. (2013). Microbe-dependent and nonspecific effects of procedures to eliminate the resident microbiota from *Drosophila melanogaster*. *Appl. Environ. Microbiol.* 79 3209–3214 10.1128/AEM.00206-13 23475620 PMC3685244

[B41] Ronshaugen M. Biemar F. Piel J. Levine M. Lai E. C. (2005). The *Drosophila* microRNA iab-4 causes a dominant homeotic transformation of halteres to wings. *Genes Dev.* 19 2947–2952 10.1101/gad.1372505 16357215 PMC1315399

[B42] Rosenberg E. Sharon G. Zilber-Rosenberg I. (2009). The hologenome theory of evolution contains Lamarckian aspects within a Darwinian framework. *Environ. Microbiol.* 11 2959–2962 10.1111/j.1462-2920.2009.01995.x 19573132

[B43] Rosenberg E. Zilber-Rosenberg I. (2011). Symbiosis and development: the hologenome concept. *Birth Defects Res. C Embryo Today* 93 56–66 10.1002/bdrc.20196 21425442

[B44] Round J. L. Mazmanian S. K. (2009). The gut microbiota shapes intestinal immune responses during health and disease. *Nat. Rev. Immunol.* 9 313–323 10.1038/nri2515 19343057 PMC4095778

[B45] Rutherford S. L. Lindquist S. (1998). Hsp90 as a capacitor for morphological evolution. *Nature* 396 336–342 10.1038/24550 9845070

[B46] Salathia N. Queitsch C. (2007). Molecular mechanisms of canalization: Hsp90 and beyond. *J. Biosci.* 32 457–463 10.1007/s12038-007-0045-9 17536165

[B47] Samakovli D. Thanou A. Valmas C. Hatzopoulos P. (2007). Hsp90 canalizes developmental perturbation. *J. Exp. Bot.* 58 3513–3524 10.1093/jxb/erm191 18057034

[B48] Sarkar B. C. Dandapathak M. Sarkar S. Banerjee T. (2013). Studies on the dynamics of two bilaterally coupled periodic gunn oscillators using melnikov techniques. *Prog. Electromagn. Res. M* 28 213–228 10.2528/PIERM12120316

[B49] Sawarkar R. Paro R. (2010). Interpretation of developmental signaling at chromatin: the Polycomb perspective. *Dev. Cell* 19 651–661 10.1016/j.devcel.2010.10.012 21074716

[B50] Schmalhausen I. I. (1949). *Factors of Evolution**: The Theory of Stabilizing Selection*. Philadelphia: Blakiston Co (reprinted in 1987 by Chicago University Press)

[B51] Sgro C. M. Wegener B. Hoffmann A. A. (2010). A naturally occurring variant of Hsp90 that is associated with decanalization. *Proc. Biol. Sci.* 277 2049–2057 10.1098/rspb.2010.0008 20200026 PMC2880099

[B52] Sharon G. Segal D. Ringo J. M. Hefetz A. Zilber-Rosenberg I Rosenberg E. (2010a). Commensal bacteria play a role in mating preference of *Drosophila melanogaster*. *Proc. Natl. Acad. Sci. U.S.A.* 107 20051–20056 10.1073/pnas.1009906107 21041648 PMC2993361

[B53] Sharon G. Segal D. Ringo J. M. Hefetz A. Zilber-Rosenberg I Rosenberg E. (2010b). Commensal bacteria play a role in mating preference of *Drosophila melanogaster*. *Proc. Natl. Acad. Sci. U.S.A.* 107 20051–20056 10.1073/pnas.1009906107 21041648 PMC2993361

[B54] Shin S. C. Kim S.-H. You H. Kim B. Kim A. C. Lee K.-A. (2011). *Drosophila* microbiome modulates host developmental and metabolic homeostasis via insulin signaling. *Science* 334 670–674 10.1126/science.1212782 22053049

[B55] Siegal M. L. Bergman A. (2002). Waddington’s canalization revisited: developmental stability and evolution. *Proc. Natl. Acad. Sci. U.S.A.* 99 10528–10532 10.1073/pnas.102303999 12082173 PMC124963

[B56] Specchia V. Piacentini L. Tritto P. Fanti L. D’Alessandro R. Palumbo G. (2010). Hsp90 prevents phenotypic variation by suppressing the mutagenic activity of transposons. *Nature* 463 662–665 10.1038/nature08739 20062045

[B57] Starr D. J. Cline T. W. (2002). A host parasite interaction rescues *Drosophila* oogenesis defects. *Nature* 418 76–79 10.1038/nature00843 12097909

[B58] Stern S. Fridmann-Sirkis Y. Braun E. Soen Y. (2012). Epigenetically heritable alteration of fly development in response to toxic challenge. *Cell Rep.* 1 528–542 10.1016/j.celrep.2012.03.012 22832276

[B59] Stern S. Snir O. Mizrachi E. Galili M. Zaltsman I. Soen Y. (2014). Reduction in maternal Polycomb levels contributes to transgenerational inheritance of a response to toxic stress in flies. *J. Physiol.* 10.1113/jphysiol.2014.271445 [Epub ahead of print] PMC4048092 24535443

[B60] Storelli G. Defaye A. Erkosar B. Hols P. Royet J. Leulier F. (2011). *Lactobacillus plantarum* promotes *Drosophila* systemic growth by modulating hormonal signals through TOR-dependent nutrient sensing. *Cell Metab.* 14 403–414 10.1016/j.cmet.2011.07.012 21907145

[B61] Stouthamer R. Breeuwer J. A. Hurst G. D. (1999). *Wolbachia pipientis*: microbial manipulator of arthropod reproduction. *Annu. Rev. Microbiol.* 53 71–102 10.1146/annurev.micro.53.1.71 10547686

[B62] Takahashi K. (2014). Influence of bacteria on epigenetic gene control. *Cell. Mol. Life Sci.* 71 1045–1054 10.1007/s00018-013-1487-x 24132510 PMC11113846

[B63] Takahashi K. Sugi Y. Nakano K. Tsuda M. Kurihara K. Hosono A. (2011). Epigenetic control of the host gene by commensal bacteria in large intestinal epithelial cells. *J. Biol. Chem.* 286 35755–35762 10.1074/jbc.M111.271007 21862578 PMC3195625

[B64] Waddington C. H. (1942). Canalization of development and the inheritance of acquired characters. *Nature* 150 563–565 10.1038/150563a0 13666847

[B65] Waddington C. H. (1957). *The Strategy of the Genes: A Discussion of Some Aspects of Theoretical Biology*. London: Allen & Unwin

[B66] Wang J. Li F. Sun R. Gao X. Wei H. Li L. J. (2013). Bacterial colonization dampens influenza-mediated acute lung injury via induction of M2 alveolar macrophages. *Nat. Commun.* 4:2106 10.1038/ncomms3106 PMC3715851 23820884

[B67] Wang Z. Klipfell E. Bennett B. J. Koeth R. Levison B. S. Dugar B. (2011). Gut flora metabolism of phosphatidylcholine promotes cardiovascular disease. *Nature* 472 57–63 10.1038/nature09922 21475195 PMC3086762

[B68] Werren J. H. (1997). Biology of *Wolbachia*. *Annu. Rev. Entomol.* 42 587–609 10.1146/annurev.ento.42.1.587 15012323

[B69] Wong C. N. A. Ng P. Douglas A. E. (2011). Low-diversity bacterial community in the gut of the fruitfly *Drosophila melanogaster*. *Environ. Microbiol.* 13 1889–1900 10.1111/j.1462-2920.2011.02511.x 21631690 PMC3495270

[B70] Wu C.-I. Shen Y. Tang T. (2009). Evolution under canalization and the dual roles of microRNAs: a hypothesis. *Genome Res.* 19 734–743 10.1101/gr.084640.108 19411598 PMC3647535

[B71] Yamanaka M. Nomura T. Kametaka M. (1977). Influence of intestinal microbes on heat production in germ-free, gnotobiotic and conventional mice. *J. Nutr. Sci.* Vitaminol. 23 221–226 10.3177/jnsv.23.221 915555

[B72] Zilber-Rosenberg I. Rosenberg E. (2008). Role of microorganisms in the evolution of animals and plants: the hologenome theory of evolution. *FEMS Microbiol. Rev.* 32 723–735 10.1111/j.1574-6976.2008.00123.x 18549407

